# Surgery for phaeochromocytomas and paragangliomas: Current practice in the United Kingdom

**DOI:** 10.1308/rcsann.2023.0054

**Published:** 2024-02-16

**Authors:** A Bojoga, SP Balasubramanian, R Mihai

**Affiliations:** ^1^ University of Medicine and Pharmacy Carol Davila, Bucharest, Romania; ^2^ Sheffield Teaching Hospitals NHS Foundation Trust, UK; ^3^ University of Sheffield, UK; ^4^ Oxford University Hospitals NHS Foundation Trust, UK

**Keywords:** Phaeochromocytoma, Variation in perioperative management, Volume–outcome correlation

## Abstract

**Introduction:**

There is wide variability in the perioperative management of phaeochromocytoma and paraganglioma (PPGL) in different centres. This study aimed to summarise the management of PPGLs as reported in the United Kingdom Registry for Endocrine and Thyroid Surgery (UKRETS) database and to determine current perioperative management of PPGLs by surveying UK clinicians.

**Methods:**

Data recorded on UKRETS from 2005 to 2021 were subjected to descriptive analyses. British Association of Endocrine and Thyroid Surgeons members were invited to participate in an open survey relating to the perioperative management of patients with PPGLs.

**Results:**

A total of 2,007 operations for PPGL from 49 participating centres were included. The median annual workload in each centre was four cases. Operations were performed predominantly laparoscopically (69%). The median length of stay (4 days) was the same in groups of surgeons stratified by volume. The survey had 29 respondents from 22 centres across the UK, and a formal protocol for perioperative management exists in 48% of the centres. Phenoxybenzamine (72%) was preferred for alpha-blockade. The practice of admitting patients for optimisation from 1 to 7 days before the day of surgery was common (62%). Central venous pressure and blood glucose monitoring were mentioned as routine intraoperative adjuncts by 72% of the responders.

**Conclusions:**

There is significant variation in the workload and perioperative management of PPGLs in the UK. This is potentially detrimental to patient outcomes and a consensus document might be beneficial to harmonise practice across the UK.

## Introduction

Phaeochromocytomas and paragangliomas (PPGL) are rare endocrine neoplasms for which surgery is the mainstay of management. These tumours are traditionally associated with significant perioperative mortality related to hypertensive crises stimulated by the surgical procedure. However, over the years, the perioperative mortality rate has dropped dramatically from around 20%^1^ or even higher in undiagnosed cases,^2^ to negligible levels more recently.^3,4^

This decrease in the mortality rate of PPGL patients is attributable to improvements in the perioperative use of alpha-blockade, surgical and anaesthetic management.^5,6^ However, the most effective practices are still debated, because current guidelines on perioperative management are largely based on traditional practice and low levels of evidence. There is wide variability in the preoperative optimisation regimens (including the type and intensity of adrenergic blockade), intraoperative drugs used and postoperative protocols implemented in different centres. In recent years, some have even questioned the need for routine perioperative alpha-blockade in modern surgical practice.^7,8^ Also, the constraints imposed on hospital beds and resources in modern healthcare (exacerbated by the COVID pandemic) raise additional questions on whether routine preoperative hospital admission and postoperative monitoring in intensive care/high dependency units (ICU/HDU) are needed for all patients. The use of different management approaches in the same cohort of patients might be a sign of either over- or underuse of medical resources. Variation may be physician and/or hospital related, driven by uncertainty regarding the optimal approach, the individual response to regulations and the accessibility of beds and facilities.^9^ Variation in practice may adversely affect clinical outcomes and understanding this may help promote interventions to enhance the quality of care.^10^

According to Hospital Episode Statistics for England (HES) data analysed in 2016, the median number of adrenal operations performed by surgeons per year in the UK was one.^11^ The reality of service provision in the UK is therefore in contrast to current Get It Right First Time recommendations^12^ that PPGLs should be operated by surgeons performing at least 12 adrenal operations per year. These recommendations are supported by empirical data demonstrating an association between volume and improved clinical outcomes in adrenal surgery.^13,14^

In the past two decades, the United Kingdom Registry for Endocrine and Thyroid Surgery (UKRETS) has been used by members of the British Association of Endocrine and Thyroid Surgeons (BAETS) to record clinical data related to endocrine surgery.^15^ The anonymised data provide insight into current practice and pathways for the delivery of endocrine surgery, and serve as a benchmark for key clinical variables.^16–18^ With just over 800 adrenal operations recorded annually on UKRETS,^15^ this audit captures the majority of, but not all adrenal operations performed in the UK. This is distinct from the HES data that capture surgery performed in all hospitals in England, regardless of whether the surgeons are members of BAETS.

This study aims to summarise the experience in the management of PPGLs as reported in the UKRETS database and to explore variability in the current perioperative management of PPGLs by surveying UK clinicians, primarily through the BAETS network.

## Methods

### Registry data analysis

Data recorded on UKRETS relating to operations for PPGLs were downloaded in anonymised form. The data set included demographics (gender and age), tumour-related data (site, size), surgical data (such as surgeon, hospital and type of surgery) and clinical outcomes (such as complications and length of stay). No identifiable patient details were available. Hospital and surgeon details were available as codes without the possibility of tracing individual clinicians or hospitals.

### Questionnaire survey

All members of BAETS were invited via the society’s mailing list to participate in an open survey relating to the perioperative management of patients with PPGLs. The survey consisted of 15 questions with multiple options and/or open answers. The survey was first sent on 22 March 2022. This was followed by a reminder on 4 May 2022 and the survey was closed on 14 May 2022. Authors’ contacts who were known to be adrenal surgeons in the UK were also included.

### Data collection and analysis

The data from both the UKRETS database and the questionnaire were available in spreadsheets and subjected to descriptive analyses. All qualitative variables are reported as frequencies and percentages. The percentages used in the reporting were rounded up to the closest integer. Quantitative data that were normally distributed are presented as means ± sd and quantitative data that were not normally distributed are presented as median and range or interquartile range (IQR). StatPlus:mac (v.8; AnalystSoft Inc.) was used for statistical analysis.

Approval for access to the UKRETS data and the questionnaire survey were obtained from the BAETS executive. It was not considered necessary to obtain informed consent from patients for the registry data, given the observational nature of the study and the lack of patient-identifiable details in the registry. Similarly, informed consent was not obtained from the clinicians involved in the survey, because participation was voluntary, and submission of responses in itself was considered as providing consent.

## Results

### UKRETS analysis of surgical management of PPGL

**Figure 1:**
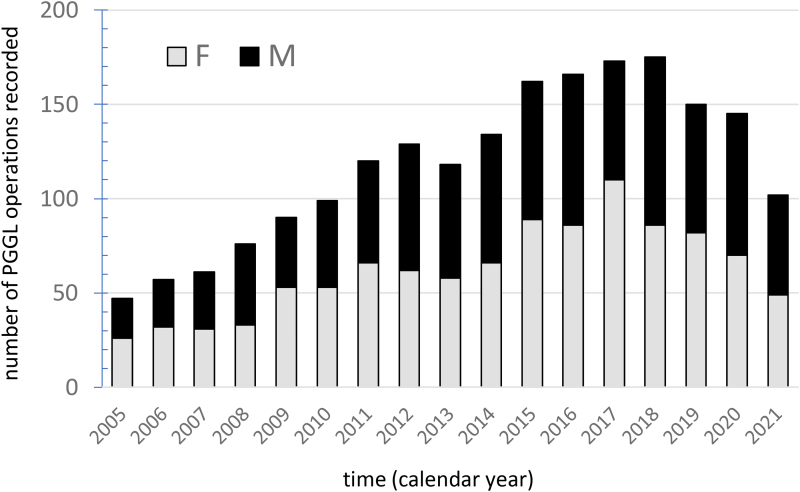
Total number of operations for phaeochromocytomas and paragangliomas (tabulated by year and gender) recorded in the United Kingdom Registry for Endocrine and Thyroid Surgery

**Figure 2:**
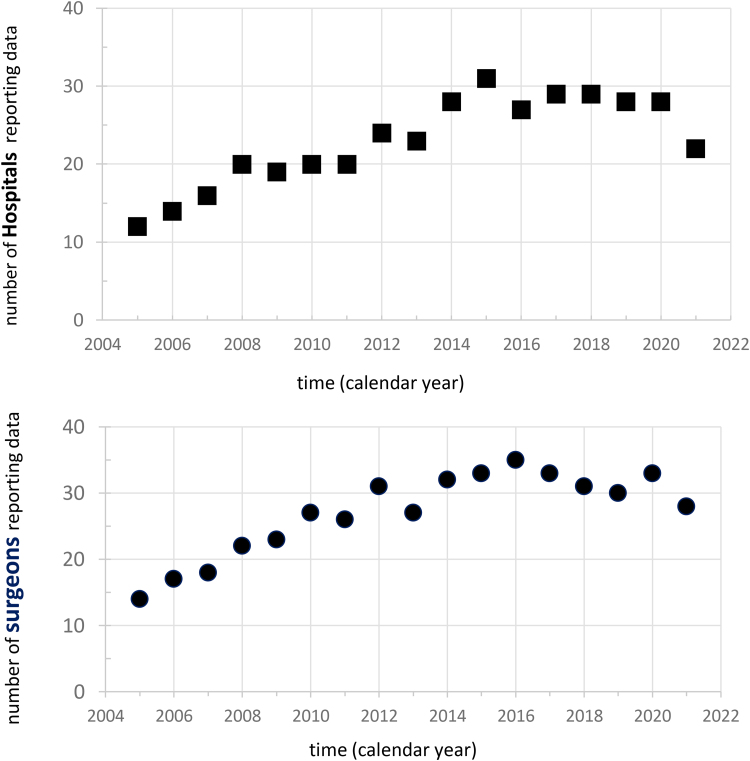
Number of hospitals and surgeons providing data over the 17 years on surgery for phaeochromocytomas and paragangliomas

**Figure 3:**
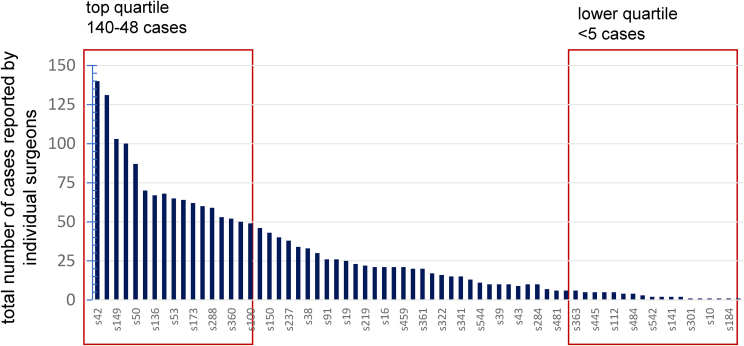
Total number of cases recorded by individual surgeons in the United Kingdom Registry for Endocrine and Thyroid Surgery during the period 2005–2021

A synopsis of recorded data in the UKRETS for PPGL surgery between 2005 and 2021 is presented in Table 1. There were a total of 2,007 cases of adrenal PPGL recorded on UKRETS with more than 150 cases per annum reported in recent years (Figure 1). Over the 17 years, data were provided from 49 hospitals (Figure 2), of which only three recorded cases in every year of the audit. The number of surgeons recording cases on UKRETS each year averaged 30 in the last decade (Figure 2) and their overall workload during the audit period varied widely, the top quartile having operated a total of over 48 patients and the lower quartile under 5 cases (Figure 3).

**Table 1:** Synopsis of recorded data on phaeochromocytoma and paraganglioma surgery in the United Kingdom Registry for Endocrine and Thyroid Surgery (2005–2021)

Characteristics of the patients with phaeochromocytoma and paraganglioma			
Gender (female : male)	1,053 : 954		
Age years (mean ± sd)			
Female	52.7 ± 17.0		
Male	53.5 ± 16.7		
Tumour localisation and surgical approach			
Left	806		
Right	920		
Bilateral	67		
Extra-adrenal	181		
Workload			
Total contribution of individual surgeons (cases)	Top quartile between 48 and 140; lower quartile <5		
Average annual workload per surgeon (cases/year)	Median 4 (range 1–20)		
Average of total experience per hospital (cases)	Median 24 (interquartile range 6–57; maximum 208)		
Surgical approach, *n* (%)			
	Total	Bilateral	Extra-adrenal
Open	341 (17)	10	128
Laparoscopic	1378 (69)	51	34
Retroperitoneoscopic	147 (7.3)	6	7
Converted to open	108 (5)	0	12
Outcomes, *n* (%)			
Readmission rate	29 (1.5)		
In-hospital death rate	7 (0.34)		

The proportion of cases operated using minimally invasive techniques increased from 72% (233/326) in the first 5 years of the audit period (2005–2009) to 82% (611/744) in the most recent 5 years (2017–2021).

Tumour size had a significant impact on the surgical approach. The median diameter of tumours in the retroperitoneoscopic, laparoscopic and open approaches was 3, 5 and 9cm respectively (Table 2). There were 108 cases converted to open surgery, in which the size of tumour ranged between 1 and 14cm (median 6cm). All but one case had a consultant as the main operator. There seemed to be no correlation between the total experience of individual surgeons and the number of conversions: a single conversion was reported by surgeons with total experience varying between 5 and 100 cases, and 6–9 conversions were reported by surgeons who had undertaken between 15 and 130 cases.

**Table 2:** Surgical approach and tumour size (mm) recorded in the United Kingdom Registry for Endocrine and Thyroid Surgery (2005–2021)

	Left side	Right side	Extra-adrenal
Posterior	28 ± 10; *n* = 39	31 ± 12; *n* = 73	
Laparoscopic	47 (5–320); *n* = 459	47 (8–188); *n* = 497	35 (10–82); *n* = 25
Open	90 (8–210); *n* = 58	90 (10–300); *n* = 91	60 (10–180); *n* = 91

Size expressed as mean ± std or median(range)

There were 11 reoperations recorded, the reasons being splenic haematoma (*n* = 1), ureteric injury (*n* = 1), infection related (*n* = 3) and major bleeding (*n* = 2; including a planned return to theatre after packing to control inferior vena cava bleeding). These cases occurred in hospitals with a median (range) total workload of 47 (26–108) cases.

The median length of stay was 4 days (IQR 3–6) during 2005–2016 and 3 days (IQR 2–5) during 2017–2021 (*p* < 0.001, Mann–Whitney *U* test). Readmissions (1.5%) were recorded after a median of 27 days after the operation; the majority related to postoperative complications (haematoma, wound infections, chest infection, portal vein thrombosis) but some were triggered by unrelated problems (fracture humerus, high stoma output, suspected epidural haematoma).

Seven in-hospital deaths were recorded from seven hospitals, after open (*n* = 4) or laparoscopic (*n* = 3) surgery.

### Survey of surgeons involved in the care of patients with PPGLs

Of 34 responses received, 29 (from 22 different centres across UK) were eligible for inclusion after duplicates and responses from centres outside the UK were excluded.

The vast majority of respondents were general/endocrine surgeons (83%) and the others were from allied subspecialties: endocrinology (1), anaesthesiology (1), urology (1), hepatobiliary and pancreatic surgery (1). The reported number of PPGL operations performed annually per centre ranged between 2 and 20 (median 9). Based on the replies, a formal protocol for perioperative management exists in 48% of the centres. Physicians were primarily involved in preoperative optimisation according to 69% of responses; four respondents said that the surgical team takes the lead in preparing patients for surgery.

**Figure 4:**
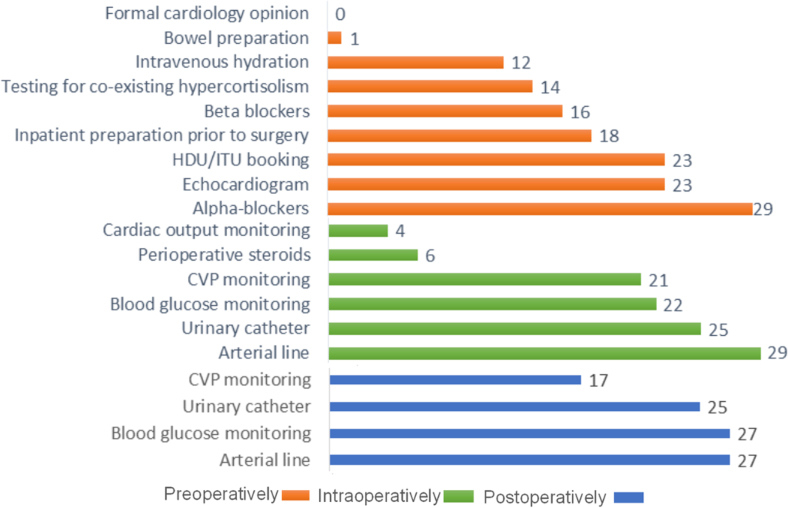
Perioperative interventions used routinely in patients with phaeochromocytoma and paraganglioma

The respondents’ routine use of different perioperative interventions is presented in Figure 4. Preoperative alpha-blockers were reported to be routinely used by all respondents, with a preference for phenoxybenzamine (72%); the rest mentioned doxazosin as the preferred choice. In total, 62% of respondents said that patients are routinely admitted for 1–2 days before surgery (8 of 13 responses) up to a maximum of 7 days preoperative admission.

From 13 responses, the most commonly reported drug used for significant hypertension during surgery was magnesium sulphate (70%) followed by nitroprusside (50%). Commonly used drugs for significant hypotension during surgery appeared to be vasopressors such as epinephrine and norepinephrine (31%) and metaraminol (5%).

## Discussion

The data from the UKRETS show that surgery for PPGL was undertaken in many hospitals with relatively low annual volume and low total workload during the study period.

Overall, none of the parameters recorded via UKRETS allowed for the demonstration of a volume–outcome correlation. Potentially, this is related to an inherent bias of relying on data recorded by motivated surgeons who are active within BAETS, and cannot be generalised to the experience of PPGL surgery in UK.

Recent GIRFT recommendations aim to restrict adrenal surgery to centres performing at least six cases per year and (ideally) refer more complex cases (phaeochromocytomas and adrenal cancer) to centres performing a minimum of 12 cases of adrenal surgeries per year. Because the data analysed were anonymised and access to UKRETS was restricted to patients operated for PPGL, the total workload of individual surgeons/centres was not available. However, based on HES data, in the most recent 5 years, there have been between 720 and 830 adrenal operations each year, performed by an average of 150 surgeons working in 60–73 hospitals. It is therefore clear that the reality of service provision is far from the standards recommended by GIRFT and this should drive the need for change in the coming years.

### Length of hospital stay and readmission rate

The median length of stay after surgery (LoS) before and after 2016 decreased significantly. This is consistent with what was reported by the latest 2021 UKRETS audit for all adrenal surgery.^15^ The median LoS of 3–4 days is low compared with the HES data set (of all adrenal surgery) with an average total LoS of 7 days.^11^

Restrictions in the nature of the data available limited an understanding of individual hospital volumes in adrenal surgery and assessment of intrahospital variation in LoS; there is likely less intrahospital variation compared with between-hospital variation.^19^

The latest UKRETS audit for adrenal surgery reports a slightly longer LoS for phaeochromocytoma surgery than for other adrenal surgery such as for Conn’s syndrome.^15^ More outcome indicators in PPGL could help assess whether this LoS is justified, or primarily based on traditional practices and historically high morbidity and/or mortality in these patients.

The recorded readmission rate in this study was low (1.5%), compared with 2.6% (confidence intervals [CI] 1.9%–3.5%) reported by the 2021 UKRETS audit for all adrenal surgery between 2016 and 2020.^15^ The HES data on adrenal surgery between 2013 and 2014 reported a higher rate of 9.8%.^11^ The lower LoS and readmission rate in the UKRETS data compared with the HES data set may be artefactual and possibly due to differences in population and ascertainment methods. UKRETS comprises data from a select group of surgeons, who are potentially higher volume than those in the rest of the country, captured by the HES. Also, both LoS and readmission rates are influenced by patient-related factors (including comorbidity) and the local environment in addition to provider volume and experience. This could explain why there was no correlation between surgery volume and LoS/postoperative stay.

### Conversions to open surgery and mortality rate

The low reported incidence of conversions from minimally invasive to open surgery in this study may have been influenced by variability in reporting – some operations may have been recorded as open without reference to the initial intention to treat those patients through a minimally invasive technique.

The in-hospital death rate (0.34%) was similar to the mortality rate at 30 days in adrenalectomy reported in 12 high-volume centres from France (0.4%).^20^ Aiming to improve mortality rates through measurement and public reporting, the National Health Service (NHS) in England has started routinely publishing outcome data for consultants in 13 specialities since 2013, as an intervention to reduce variation in surgical practice.^21^ However, small patient samples per physician, such as in PPGL surgery, make estimates more prone to a chance event and judgements of physician performance less reliable.^22^ However, increasing transparency and accountability is an effective intervention for decreasing variation in medical practice.^10^

### Perioperative management practices

The survey of BAETS members provided an interesting insight into current UK practice concerning the perioperative management of PPGLs. A formal protocol for perioperative management exists in fewer than half of the centres, leading to inherent variation. Preoperative medical optimisation with alpha-blockade was used by all responders and is based on the historical low-quality evidence that it reduces the risk of haemodynamic instability and might improve clinical outcomes.^6,23^ The most commonly used alpha-adrenergic blocker remains phenoxybenzamine despite recent challenges in securing access to a rarely used drug. Mirroring the experience reported from hospitals outside the UK, doxazosin is the drug preferred by a significant minority of responders to this survey. However, the first recent randomised control trial in patients scheduled for resection of a PPGL^3^ reported no difference in terms of haemodynamic instability between selective (phenoxybenzamine) and non-selective (doxazosin) alpha-blockers, and both are recommended by current guidelines.^24^

Almost half of the responders admit patients for inpatient preparation preoperatively, probably driven by concerns related to the risk of orthostatic hypotension associated with non-selective alpha-blockers. This practice is not unique to the UK, as in some university medical centres in the Netherlands patients are also admitted routinely to hospital.^25^

An additional reason for preoperative admission has been the need for intravenous hydration, reportedly used routinely by almost half of the responders (41%). However, a recent cohort study compared patients who were rehydrated with ≥2,000ml daily for ≥2days before surgery with patients who did not receive any intravenous rehydration preoperatively and concluded that this practice did not improve perioperative haemodynamics.^26^

Most responders booked a critical care (ICU/HDU) bed for postoperative monitoring following adrenalectomy for PPGLs, as recommended by current guidelines.^24^ Some challenge these recommendations, suggesting that most patients could be safely discharged to the ward after a few hours of monitoring,^27^ an approach that could reduce the cost of in-hospital care by 34%.^28^

There is controversy in many aspects of the preoperative, intraoperative and postoperative management of patients with PPGLs. Unwarranted variation in care is undesirable because some patients do not get the interventions they need or get more than they need.^9^ Reducing this is important to all shareholders concerned about effectiveness, efficiency and equity in healthcare.^10^ Current guideline recommendations based mainly on local practices may be ineffective and increase costs. However, clinical trials are difficult to conduct for many reasons: surgical treatment involved, ethical concerns raised by the controversies of perioperative preparations, and not least, the rarity of the disease with a very low morbidity–mortality. Medical registries may be a source of reliable data^29^ which may help mitigate professional uncertainty, increasing transparency and accountability both at an individual and organisational level, decrease variation in medical practice, and improve the quality of care.

### Limitations of the study

There are significant limitations to this study. Recording data in UKRETS is voluntary, and the data are susceptible to selective reporting bias and are not formally validated. Generalisability is limited because many adrenal surgeons in the UK are not members of BAETS, and their workload and individual outcomes remain difficult to quantify. Making adrenal key performance indicators compulsory in UKRETS, as suggested by the latest BAETS audit,^15^ as well as public reporting and feedback on physician and hospital outcomes, would probably alleviate reporting bias and improve the missing data rates. The responders of the survey were known to have large-volume adrenal practice and might not reflect the practice in smaller units. Clinicians in other related specialities such as endocrinology and anaesthesiology involved in perioperative management were not invited to participate in this survey.

A further limitation of this study is the lack of long-term outcome data. This is justified and cannot be overcome, because UKRETS relies on anonymous data, with the removal of the date of birth of each patient in response to General Data Protection Regulation concerns (replaced with the age three years after data submission).

## Conclusions

Despite the limitations described, there is evidence of significant variation relating to perioperative management of PPGLs in the UK, partly because current recommendations are based on low-level evidence. Large randomised control trials to establish the most appropriate management strategies are unlikely to be undertaken, but observational data from registries with data validation may be a solution. Questions remain over whether a consensus document is needed to address this; unwarranted variation is potentially detrimental to patients’ outcomes and effective interventions to decrease it, targeting both doctors and hospitals, need to be explored.
